# Pericoronary adipose tissue radiomics enhances prediction of major adverse cardiovascular events beyond CCTA-derived functional parameters in coronary atherosclerosis

**DOI:** 10.3389/fcvm.2026.1833189

**Published:** 2026-05-12

**Authors:** Zhenye Wang, Zhijing Wu, Milan Cao, Lili Zhao, Guojiang Zhang, Shan Wu, Xiong Zhang, Jiwei Ning, Yanhua Zhang, Junqin Wang, Lei Yin, Qiang Wang, Zhigao Xu

**Affiliations:** 1 Department of Radiology, The Third People's Hospital of Datong, Datong, China; 2 First Clinical Medical College, Graduate School, Changzhi Medical College, Changzhi, China; 3 Department of Science and Education, The Third People's Hospital of Datong, Datong, China; 4 Department of Cardiology, The Third People's Hospital of Datong, Datong, China; 5 Department of Radiology, Shanxi Bethune Hospital, Taiyuan, China; 6 Department of Electronic and Information Engineering, Taiyuan University of Science and Technology, Taiyuan, China; 7 Department of Clinical Laboratory, The Third People's Hospital of Datong, Datong, China; 8 Department of Computer and Network Engineering, Shanxi Datong University, Datong, China

**Keywords:** coronary atherosclerosis, machine learning, major adverse cardiovascular events, pericoronary adipose tissue, radiomics

## Abstract

**Objective:**

To explore the predictive value of a combined model integrating perivascular coronary adipose tissue (PCAT) radiomics features and coronary computed tomography angiography (CCTA)-derived functional parameters for major adverse cardiovascular events (MACE) in patients with Coronary Atherosclerosis (CAS).

**Methods:**

This retrospective study enrolled 171 CAS patients who underwent CCTA at Datong Third People's Hospital between November 2020 and September 2022 and stratified them into a MACE-positive group (*n* = 72) and a MACE-negative group (*n* = 99) based on the occurrence of MACE. Using support vector machine (SVM) and Gaussian process regression (GPR) algorithms, we constructed four MACE prediction models: two models relying on CCTA-derived functional parameters (stenosis severity and CT-FFR), and two combined models integrating these parameters with the radiomics score (Rad-score). Model performance was evaluated using the area under the receiver operating characteristic curve (AUC), F1-score, Delong test, calibration curves, and decision curve analysis (DCA).

**Results:**

Among the CCTA-derived functional parameters, CT-derived fractional flow reserve (CT-FFR) and coronary stenosis severity emerged as independent predictors of MACE in patients with CAS (both *P* < 0.05). Models integrating CCTA-derived functional parameters with the radiomics score (Rad-score) demonstrated superior predictive performance compared with models relying solely on CCTA-derived functional parameters. Specifically, the mean AUC for SVM and GPR models based exclusively on CCTA-derived functional parameters were 0.742 and 0.737, respectively. In contrast, the mean AUCs for the corresponding combined SVM and GPR models both increased to 0.803. Notably, the combined GPR model achieved the highest mean F1-score (0.686). The DeLong test confirmed that the combined models significantly outperformed the CCTA-only models in both the training and testing sets (all *P* < 0.05). Calibration curves revealed the best goodness-of-fit for the combined GPR model, and DCA indicated that this model provided the greatest net clinical benefit across a broad range of decision thresholds.

**Conclusion:**

PCAT radiomics features can enhance the predictive performance of models based on CCTA-derived functional parameters for MACE in CAS patients. Notably, the combined GPR model exhibits optimal predictive accuracy and clinical utility.

## 1 Introduction

Cardiovascular diseases encompass a spectrum of disorders affecting the heart and blood vessels, including coronary artery disease (CAD), cerebrovascular disease, rheumatic heart disease, and other related conditions. In 2019, the World Health Organization (WHO) identified CVDs as the leading global cause of mortality over the preceding two decades (1). Among these, CAD stands as one of the primary cardiovascular disorders contributing to death and disability worldwide (2). With the annual increase in the incidence of CAD, the prevalence of major adverse cardiovascular events (MACE)—defined as cardiac death, unplanned revascularization, fatal or non-fatal myocardial infarction, and stroke—has correspondingly risen, posing a significant threat to global public health (3).

The 2024 Guidelines of the European Society of Cardiology (ESC) recommend coronary computed tomography angiography (CCTA) as a first-line non-invasive imaging modality for the evaluation of coronary atherosclerosis (CAS), given its advantages as a non-invasive anatomical imaging tool (4). CCTA images contain abundant anatomical, morphological, and physiological information: they not only allow for the assessment of coronary artery stenosis severity but also enable the calculation of CT-derived fractional flow reserve (CT-FFR) via computational fluid dynamics methods, thereby facilitating the evaluation of myocardial ischemia extent (5). Pericoronary adipose tissue (PCAT)—defined as fat deposition surrounding the coronary arteries and a component of epicardial adipose tissue—exhibits distinct pathophysiological features and plays a specific role in the progression of coronary atherosclerosis (6). Research (7) has established that the signaling crosstalk between perivascular adipose tissue (PVAT) and coronary arteries is bidirectional. In the pathogenesis and progression of CAS, PVAT may drive vascular inflammation via outside-to-inside signaling, while mediators within the vessel wall can modulate PVAT through inside-to-outside signaling—endowing PVAT with amplified pro-inflammatory properties. On CCTA images, a heightened inflammatory state is reflected by increased fat attenuation; however, this metric only captures the global status of PVAT and fails to characterize higher-dimensional features.

With the rapid advancement of interdisciplinary integration, radiomics has emerged as an innovative approach for PVAT research. By leveraging automated algorithms to extract high-dimensional quantitative features from medical images, radiomics uncovers microstructural information that is not readily detectable via conventional visual assessment, thus demonstrating substantial potential in cardiovascular medicine (8). Nevertheless, studies integrating PVAT-based radiomics with CCTA-derived functional parameters to predict MACE in CAS patients remain under-explored. Accordingly, this study aims to develop a combined predictive model that integrates both PVAT radiomic features and CCTA functional parameters, and to evaluate its performance in predicting MACE in CAS patients. The goal is to provide a robust tool for the early identification of MACE risk, thereby informing clinical decision-making.

## 2 Materials and methods

### 2.1 Participants

A total of 237 patients with CAS who met the eligibility criteria at the Third People's Hospital of Datong were retrospectively enrolled between November 2020 and September 2022. Following rigorous screening according to predefined inclusion and exclusion criteria, 171 patients were ultimately included in the final analysis—comprising 88 males and 83 females, aged 35–82 years (mean age: 61 years). Based on the occurrence of MACE during follow-up, we stratified participants into the MACE-positive group (*n* = 72) and MACE-negative group (*n* = 99). MACE was defined as the presence of at least one of the following endpoints: (1) readmission for unstable angina pectoris with objective evidence of myocardial ischemia at admission, including elevated myocardial enzymes, abnormal electrocardiogram, or echocardiographic abnormalities; (2) unplanned revascularization for unstable angina pectoris; (3) non-fatal myocardial infarction; (4) cardiac death.

Inclusion criteria were as follows: (1) Underwent CCTA, and the results were assessed as Coronary Artery Disease-Report and Data System (CAD-RADS) 2.0 grade 2–3 (9), indicating coronary artery stenosis of 25%–69% in one or more vessels; (2) Received medical therapy alone without prior revascularization before enrollment; (3) Complete imaging data were available; (4) Complete medical history records and follow-up outcomes were obtained. Exclusion criteria included: (1) Inability to identify the culprit vessel (*n* = 26); (2) History of prior myocardial infarction (*n* = 3); (3) History of prior stent implantation (*n* = 5); (4) Suboptimal image quality precluding diagnostic analysis (*n* = 15); (5) Missing clinical data (*n* = 12); [6] Occurrence of MACE within 3 months after enrollment (*n* = 5).

### 2.2 Baseline clinical data collection and follow-up

We extracted baseline clinical data for all enrolled patients from the hospital's electronic medical record system and conducted subsequent follow-up predominantly through structured telephone interviews. The follow-up interval began on the date of the initial CCTA examination and ended on September 30, 2024. The primary endpoint was the first occurrence of MACE or the prespecified follow-up termination date—whichever came earlier. Of the 171 enrolled patients, 72 (42.1%) experienced at least one MACE during follow-up. MACE comprised: (1) 31 hospitalizations for unstable angina pectoris—22 with elevated cardiac biomarkers and 9 with electrocardiographic or echocardiographic abnormalities upon admission; (2) 34 revascularization procedures for unstable angina pectoris, all performed via percutaneous coronary intervention (PCI); and (3) 7 cases of non–ST-segment elevation myocardial infarction (NSTEMI). A total of 23 baseline clinical variables were collected in this study, which are detailed in Table 1.

**Table 1:** Univariate analysis and collinearity testing of clinical baseline characteristics in CAS patients.

Item	Data Type	Normality Test Result		Univariate Regression Test Method	Value		*P*-value	*t*/U/OR/*x^2^*	Collinearity Check (VIF)
		MACE=0*	MACE=1		MACE=0	MACE=1			
Age	Continuous	Normal (*p* = 0.16)	Normal (*p* = 0.40)	Independent t-test	60.84 ± 9.55	60.93 ± 10.06	0.95	−0.06	1.25
TC	Continuous	Non-normal (*p* = 0.00)	Normal (*p* = 0.17)	Mann–Whitney U test	4.53 (3.83, 5.57)	4.76 ± 1.14	0.68	3429.50	22.04
LDL	Continuous	Non-normal (*p* = 0.00)	Normal (*p* = 0.21)	Mann–Whitney U test	2.71 (1.89, 3.38)	2.85 ± 0.88	0.55	3372.50	13.95
**HDL**	**Continuous**	**Normal (*p*** **=** **0.09)**	**Non-normal (*p*** **=** **0.00)**	**Mann–Whitney U test**	**1.23** **±** **0.28**	**1.10 (0.92, 1.29)**	**0**.**02**	**4323**.**00**	**1**.**56**
TG	Continuous	Non-normal (*p* = 0.00)	Non-normal (*p* = 0.00)	Mann–Whitney U test	1.61 (1.17, 2.24)	1.67 (1.29, 2.35)	0.35	3266.50	4.06
Calcium Score	Continuous	Non-normal (*p* = 0.00)	Non-normal (*p* = 0.00)	Mann–Whitney U test	42.98 (3.53, 92.36)	70.61 (3.42, 169.32)	0.09	3030.50	2.51
Stenosis	**Continuous**	**Normal (*p*** **=** **0.25)**	**Non-normal (*p*** **=** **0.00)**	**Mann–Whitney U test**	**0.45** **±** **0.10**	**0.55 (0.48, 0.64)**	**0**.**00**	**1971**.**50**	**1**.**83**
CT-FFR	**Continuous**	**Non-normal (*p*** **=** **0.00)**	**Non-normal (*p*** **=** **0.00)**	**Mann–Whitney U test**	**0.87 (0.84, 0.90)**	**0.83 (0.79, 0.88)**	**0**.**00**	**4842**.**50**	**1**.**50**
Culprit Plaque Volume (mm^3^)	Continuous	Non-normal (*p* = 0.00)	Non-normal (*p* = 0.00)	Mann–Whitney U test	61.41 (41.78, 109.91)	157.15 (78.09, 254.34)	0.00	1855.00	5.45
Culprit Plaque Length (mm)	Continuous	Non-normal (*p* = 0.00)	Non-normal (*p* = 0.00)	Mann–Whitney U test	15.00 (11.55, 24.00)	24.75 (15.97, 41.33)	0.00	2275.00	5.06
Calcified Component (%)	Continuous	Non-normal (*p* = 0.00)	Non-normal (*p* = 0.03)	Mann–Whitney U test	63.14 (44.58, 85.40)	52.25 (32.44, 73.05)	0.01	4408.50	24069958.47
Lipid Component (%)	Continuous	Non-normal (*p* = 0.00)	Non-normal (*p* = 0.00)	Mann–Whitney U test	3.97 (0.67, 8.08)	7.12 (2.76, 12.64)	0.00	2630.00	2615065.47
Fibro-Lipid Component (%)	Continuous	Non-normal (*p* = 0.00)	Normal (*p* = 0.37)	Mann–Whitney U test	23.98 (9.52, 39.41)	32.22 ± 17.21	0.02	2823.00	11868605.34
Fibrous Component (%)	Continuous	Non-normal (*p* = 0.02)	Non-normal (*p* = 0.00)	Mann–Whitney U test	5.80 (3.33, 6.89)	5.48 (4.38, 6.95)	0.66	3423.50	261828.08
Gender	**Categorical**	**Not Applicable**	**Not Applicable**	**Fisher's exact test**	**0: 61, 1: 38**	**0: 22, 1: 50**	**0**.**00**	**3**.**65**	**2**.**19**
History of Hypertension	Categorical	Not Applicable	Not Applicable	Fisher's exact test	0: 47, 1: 52	0: 25, 1: 47	0.12	1.70	1.29
History of Diabetes	Categorical	Not Applicable	Not Applicable	Fisher's exact test	0: 74, 1: 25	0: 46, 1: 26	0.13	1.67	1.24
Family History of CVD	Categorical	Not Applicable	Not Applicable	Fisher's exact test	0: 87, 1: 12	0: 62, 1: 10	0.82	1.17	1.19
Smoking History	**Categorical**	**Not Applicable**	**Not Applicable**	**Fisher's exact test**	**0: 71, 1: 28**	**0: 39, 1: 33**	**0**.**024**	**2**.**15**	**2**.**80**
Drinking History	**Categorical**	**Not Applicable**	Not Applicable	**Fisher's exact test**	**0: 75, 1: 24**	**0: 40, 1: 32**	**0**.**00**	**2**.**50**	**2**.**77**
Hyperlipidemia	Categorical	Not Applicable	Not Applicable	Fisher's exact test	0: 7, 1: 92	0: 4, 1: 68	0.76	1.29	1.13
Culprit Vessel	Categorical	Not Applicable	Not Applicable	Chi-square test	1: 76, 2: 4, 3: 19	1: 45, 2: 5, 3: 22	0.13	4.11	1.24
Number of High-Risk Features	**Categorical**	**Not Applicable**	**Not Applicable**	**Chi-square test**	**0: 15, 1: 3, 2: 34, 3: 30, 4: 17**	**0: 6, 1: 5, 2: 11, 3: 25, 4: 25**	**0**.**01**	**14**.**18**	**1**.**89**

CAS, coronary atherosclerosis; MACE, major adverse cardiovascular events; TC, total cholesterol; LDL, low-density lipoprotein; HDL, high-density lipoprotein; TG, triglyceride; CT-FFR, CT-derived fractional flow reserve; CVD, Cardiovascular diseases.

* MACE = 0: MACE-negative group, MACE = 1: MACE-positive group; Bold values indicate statistically significant variables (*P* < 0.05).

### 2.3 Identification of culprit vessels

Patients with CAS who developed abnormalities in cardiac enzymes, electrocardiography, or echocardiography, or underwent unplanned revascularization during follow-up were defined as MACE-positive patients (10, 11). The culprit vessel was determined according to the following criteria: (1) If baseline measurement showed a single coronary artery with stenosis >50%, that vessel was directly considered the culprit vessel; (2) If multiple vessels had baseline stenosis >50%, the vessel that actually received unplanned revascularization was designated as the culprit vessel; (3) Regardless of the degree of stenosis, when wall motion abnormality was detected by electrocardiography or echocardiography, the vessel supplying the corresponding abnormal region was identified as the culprit vessel. For MACE-negative patients, the vessel with the most severe stenosis was selected as the culprit vessel. All culprit vessels in this study were determined by senior cardiologists with expertise in coronary angiography, based on comprehensive analysis of patients' laboratory test results, surgical records, electrocardiograms, and echocardiograms.

### 2.4 Image acquisition and data extraction

We acquired all CCTA images using a Siemens third-generation dual-source computed tomography (CT) scanner (SOMATOM Force CT, Siemens Healthineers, Germany) with retrospective electrocardiogram gating. Scanning covered the region from 1 cm above the aortic arch to 1 cm below the diaphragmatic surface of the heart, with the acquisition time window set at 30%–80% of the R-R interval. Iodixanol (320 mgI/mL Visipaque, GE Healthcare, USA) was administered as the contrast agent via a bolus-tracking technique: the region of interest (ROI) was placed in the ascending aorta, and scanning automatically triggered upon reaching a preset attenuation threshold of 120 Hounsfield units (Hu).Tube current was dynamically adjusted based on patient body habitus using an automatic exposure control system, with ECG gating applied for further optimization. The CT system automatically reconstructed images of the optimal diastolic and systolic phases with a slice thickness of 0.625 mm and the BV40 convolution kernel. All raw data were uploaded to the uAI Research Portal platform (https://urp.united-imaging.com) (12).

Image post-processing and extraction of target imaging parameters were performed on the uAI Research Portal. Two senior radiologists (with ≥10 years of clinical experience) independently selected the optimal phase for image feature assessment; any discrepancies were resolved through consensus discussion. We assessed the number of high-risk plaque features by integrating visual interpretation with machine learning–assisted analysis. Plaque volume, length, and lipid content percentage were quantified automatically using the platform's embedded machine learning module, followed by manual verification to ensure measurement accuracy.

### 2.5 PCAT segmentation

PCAT segmentation and delineation were performed using the uAI Research Portal (https://urp.united-imaging.com). For the culprit vessel, we defined the PCAT region as the adipose tissue extending outward from the vessel's outer wall by a distance equal to one vessel diameter, with a fat attenuation threshold set at −190 to −30 Hu (13). The analyzed segments of each coronary artery were specified as follows: left anterior descending artery (LAD): 0–40 mm; left circumflex artery (LCX): 0–40 mm; right coronary artery (RCA): 10–50 mm.

### 2.6 Radiomics feature extraction for PCAT

On the uAI Research Portal, all images were first normalized using the mean variance normalization method, followed by the extraction of radiomics features. A total of 2,264 radiomics features were extracted from PCAT (from 15 filters: Original, BoxMean, AdditiveGaussianNoise, BinomialBlurImage, CurvatureFlow, BoxsigmaImage, LoG, wavelet, Normalize, LaplacianSharpening, DiscreteGaussian, Mean, SpeckleNoise, RecursiveGaussian, ShotNoise), including 450 first-order features, 14 shape features, and 1,800 texture features.

### 2.7 PCAT radiomics feature selection

**Figure 1:**
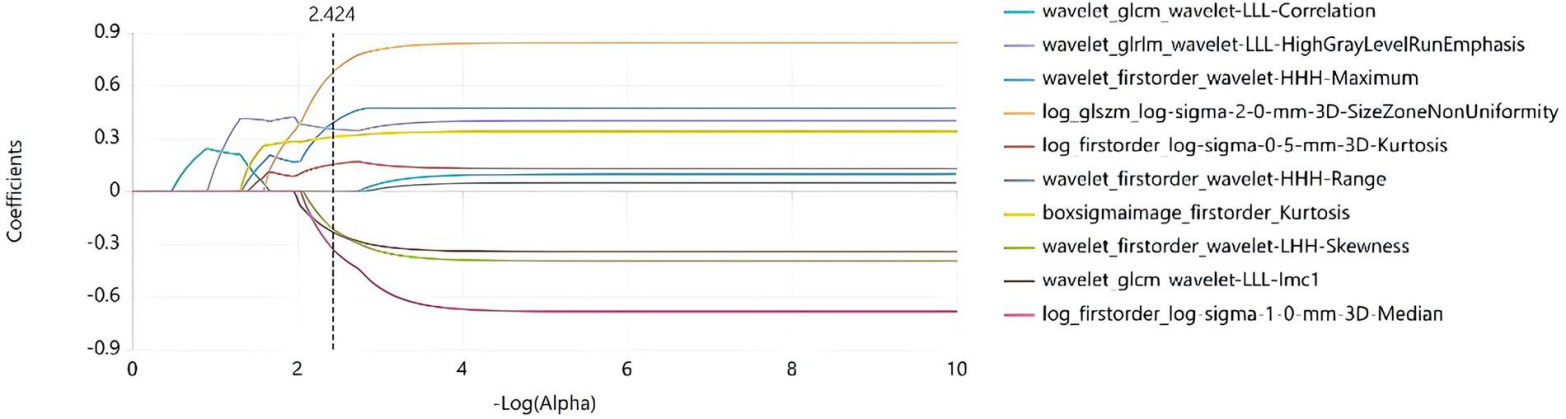
Lasso coefficient path plot.

**Figure 2:**
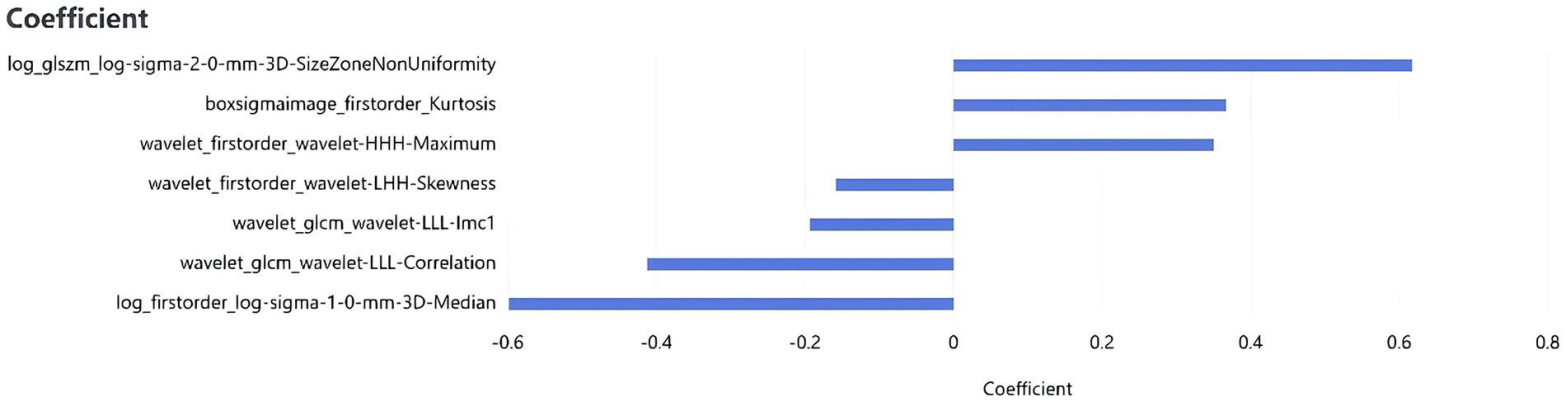
The seven selected radiomic features.

**Figure 3:**
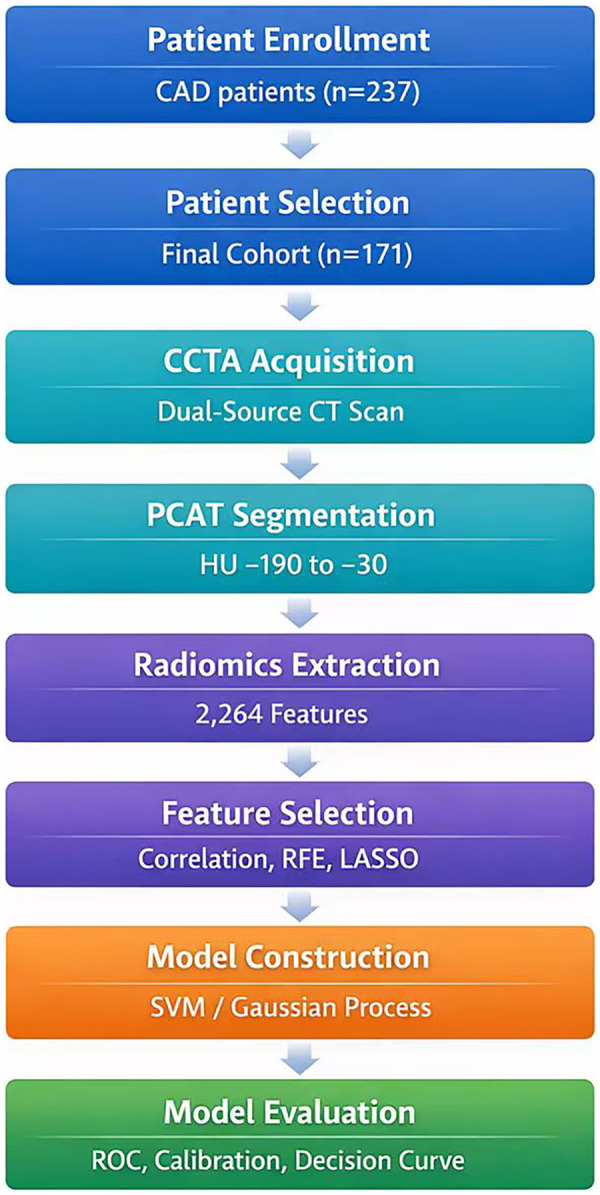
Flowchart of the radiomics analysis. CAS, coronary atherosclerosis; CCTA, coronary computed tomography angiography; PCAT, perivascular coronary adipose tissue.

There are 72 MACE-positive samples and 99 MACE-negative samples in this study. To adhere to the “10 events per variable” (10 EPV) principle, which recommends at least 10 outcome events for each predictor variable to avoid overfitting, the number of predictor variables in the final model was limited to no more than 7, given the 72 MACE events. To mitigate the risk of model overfitting and assess the stability of selected features, we employed a feature selection strategy based on 5-fold cross-validation (random seed: 42). Prior to analysis, max-abs scaling (dividing by the maximum absolute value to scale features to the range [-1, 1]) was applied to eliminate dimensional discrepancies among radiomics features. We then selected optimal radiomics features using a sequential approach: first, correlation coefficient analysis to remove highly correlated features; second, recursive feature elimination (RFE) to rank feature importance; and finally, least absolute shrinkage and selection operator (Lasso) regression to identify the most predictive features and their coefficients. Ultimately, 7 radiomics features were chosen to construct a single radiomics score (Rad-score) by linear combination using the Lasso coefficients. This Rad-score was then combined with clinical predictors in the final predictive model, ensuring the total number of variables remained within the 10 EPV limit. A Rad_score formula was derived based on the selected features and their corresponding regression coefficients, defined as: Rad_score = 0.7296135 + *Σ* (featureᵢ × coefficientᵢ). Details are provided in Figures 1, 2. A flowchart of the radiomics analysis is provided in Figure 3.

### 2.8 Model construction

Among the 171 participants included in this study, 72 experienced MACE, leading to a relatively small MACE cohort and class imbalance. To improve model generalizability and reduce overfitting, we performed hyperparameter tuning using grid search with 5-fold cross-validation. Four MACE prediction models were developed using support vector machine (SVM) and Gaussian process regression (GPR): (1) SVM model based on clinical independent predictors; (2) GPR model based on clinical independent predictors; (3) SVM model based on combined clinical-imaging data; and (4) GPR model based on combined clinical-imaging data.

Model performance was evaluated using receiver operating characteristic (ROC) curves. For each model, we calculated the mean area under the ROC curve (AUC), sensitivity, specificity, accuracy, precision, and F1 score across 5-fold cross-validation. The Delong test was used to compare the predictive efficacy between models. Calibration curves assessed the agreement between predicted probabilities and observed event rates. Decision curve analysis (DCA) quantified net benefit across a range of risk thresholds to evaluate clinical utility. We selected the optimal model based on overall performance metrics and used permutation importance to rank feature contributions, thereby improving model interpretability. The specific data preprocessing parameters, model hyperparameter settings, and custom algorithms are shown in Supplementary Table S1.

### 2.9 Statistical analysis

Statistical analyses were performed using Python 3.9. Normality of continuous variables was assessed via normality tests; variables with a *p*-value > 0.05 were deemed to follow a normal distribution. For univariate analysis of continuous variables: normally distributed variables were compared using the independent-samples *t*-test and reported as mean ± standard deviation (SD); non-normally distributed variables were analyzed using the Mann–Whitney U test and reported as median. For univariate analysis of categorical variables: binary variables were evaluated using Fisher's exact test, while multi-categorical variables were tested using the chi-square test. All tests were two-tailed, and a *p*-value < 0.05 was considered statistically significant.

## 3 Results

### 3.1 Clinical baseline characteristics of patients with CAS

In this study, we first performed univariate analysis on 23 clinical indicators across the MACE-positive and MACE-negative groups. A total of 12 indicators significantly associated with MACE (*p* < 0.05). Collinearity diagnostics were then conducted, and indicators with a VIF > 5 were excluded. Based on these results, 7 indicators were included in a multivariate binary logistic regression analysis, which yielded 3 independent predictors of MACE from clinical characteristics: stenosis severity, CT-FFR, and sex. Additionally, univariate analysis revealed a statistically significant difference in Rad_score between the MACE-positive and MACE-negative groups (*p* < 0.05; see Tables 1, 2). Finally, two CCTA-derived functional parameters—stenosis severity and CT-FFR—were selected to construct a clinical prediction model for MACE in CAS patients. In accordance with the study aim of evaluating CT-derived biomarkers, clinical variable (sex) was intentionally not included in this model. To develop a combined clinical-imaging prediction model, Rad_score was incorporated into the model alongside stenosis severity and CT-FFR.

**Table 2:** Multivariable regression analysis of clinical baseline characteristics.

Item	OR (95% CI)	*P*-value
const	61.625 (0.074–51462.087)	0.230
HDL	0.355 (0.094–1.343)	0.127
Stenosis	**1.070 (1.031–1.109)**	**0**.**000**
CT-FFR	**0.919 (0.860–0.982)**	**0**.**012**
Number of High-Risk Features	0.998 (0.731–1.362)	0.988
Gender	**3.770** (**1.494–9.513)**	**0**.**005**
Smoking History	0.571 (0.180–1.810)	0.341
Drinking History	1.763 (0.552–5.633)	0.339

HDL, high-density lipoprotein; CT-FFR, CT-derived fractional flow reserve.

Bold values indicate statistically significant variables (*P* < 0.05).

### 3.2 Performance comparison of MACE prediction models in patients with CAS

Cross-validation results demonstrate that: CCTA-derived model 1, which employed a SVM approach based on coronary artery stenosis and CT-FFR, achieved an AUC of 0.742 and an F1 score of 0.316 for predicting MACE. CCTA-derived model 2, utilizing GPR with the same clinical features, yielded an AUC of 0.737 and an F1 score of 0.566. In comparison, the combined models, which integrated radiomic features (Rad-score) with clinical indicators, demonstrated improved predictive performance. Combined Model 1 (SVM based on stenosis + CT-FFR + Rad-score) achieved an AUC of 0.803 and an F1 score of 0.502, while Combined Model 2 (GPR based on the same features) also reached an AUC of 0.803 but with a higher F1 score of 0.686.

**Figure 4:**
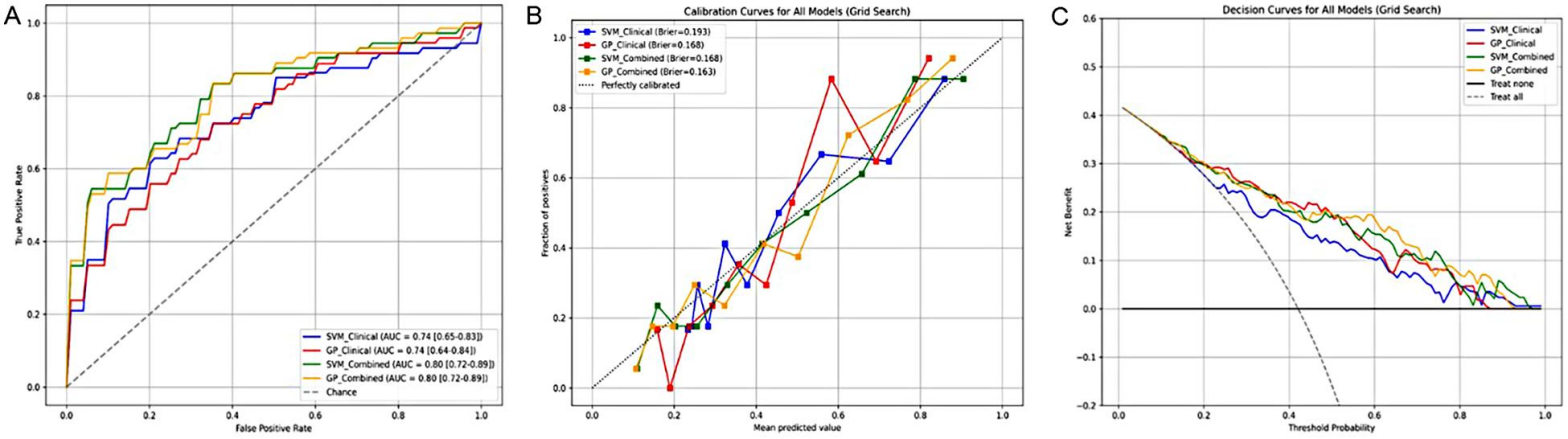
Performance evaluation of four machine learning models for predicting MACE. **(A)** ROC Curves for Four MACE Prediction Models; **(B)** Calibration Curves for Four MACE Prediction Models; **(C)** Decision Curves for Four MACE Prediction Models. SVM, support vector machine; GP, Gaussian process regression.

Delong test results revealed that the differences in AUC between the CCTA-derived models and the combined models were statistically significant (*p* < 0.01). In contrast, no significant differences were observed between CCTA-derived model 1 and CCTA-derived model 2, nor between Combined Model 1 and Combined Model 2 (*p* > 0.05). These findings indicate that the inclusion of radiomic features significantly enhances predictive performance over clinical features alone. Further multi-parameter assessment showed that Combined Model 2 (AUC: 0.803, sensitivity: 0.628, specificity: 0.867, accuracy: 0.765, precision: 0.773, F1-score: 0.686) was superior to Combined Model 1 (AUC: 0.803, sensitivity: 0.451, specificity: 0.877, accuracy: 0.697, precision: 0.570, F1-score: 0.502) (see Tables 3, 4 and Figure 4A).

**Table 3:** Performance of four MACE prediction models.

Model	Sensitivity	Specificity	Accuracy	Precision	F1-Score	AUC	95% CI	Brier Score
SVM_Clinical	0.251	0.930	0.643	0.433	0.316	0.742	0.647−0.835	0.193
GP_Clinical	0.502	0.827	0.690	0.677	0.566	0.737	0.637–0.839	0.168
SVM_Combined	0.451	0.877	0.697	0.570	0.502	0.803	0.721–0.887	0.168
GP_Combined	0.628	0.867	0.766	0.773	0.686	0.803	0.722–0.887	0.163

SVM, support vector machine; GP, Gaussian process regression.

**Table 4:** Delong's test for four MACE prediction models.

Model Comparison	Z Statistic	*P*-value
SVM_Clinical vs. GP_Clinical	−0.328	0.743
SVM_Clinical vs. SVM_Combined	−3.517	0.000
SVM_Clinical vs. GP_Combined	−3.750	0.000
GP_Clinical vs. SVM_Combined	−2.947	0.003
GP_Clinical vs. GP_Combined	−3.030	0.002
SVM_Combined vs. GP_Combined	0.474	0.636

SVM, support vector machine; GP, Gaussian process regression.

Calibration curves demonstrated that combined models were more closely aligned with the ideal curve than CCTA-derived models; notably, Combined Model 2 had the highest goodness-of-fit (Brier score = 0.163), indicating optimal calibration and the most accurate probability prediction (Figure 4B). Clinical decision curves showed that when the risk threshold ranged from 30% to 80%, all models yielded higher net benefits than the “treat all” or “treat none” strategies, confirming their clinical utility. Among these, Combined Model 2 achieved the highest clinical net benefit and thus the best clinical decision-making value (Figure 4C).

### 3.3 Interpretability analysis of the MACE prediction model in CAS patients

**Figure 5:**
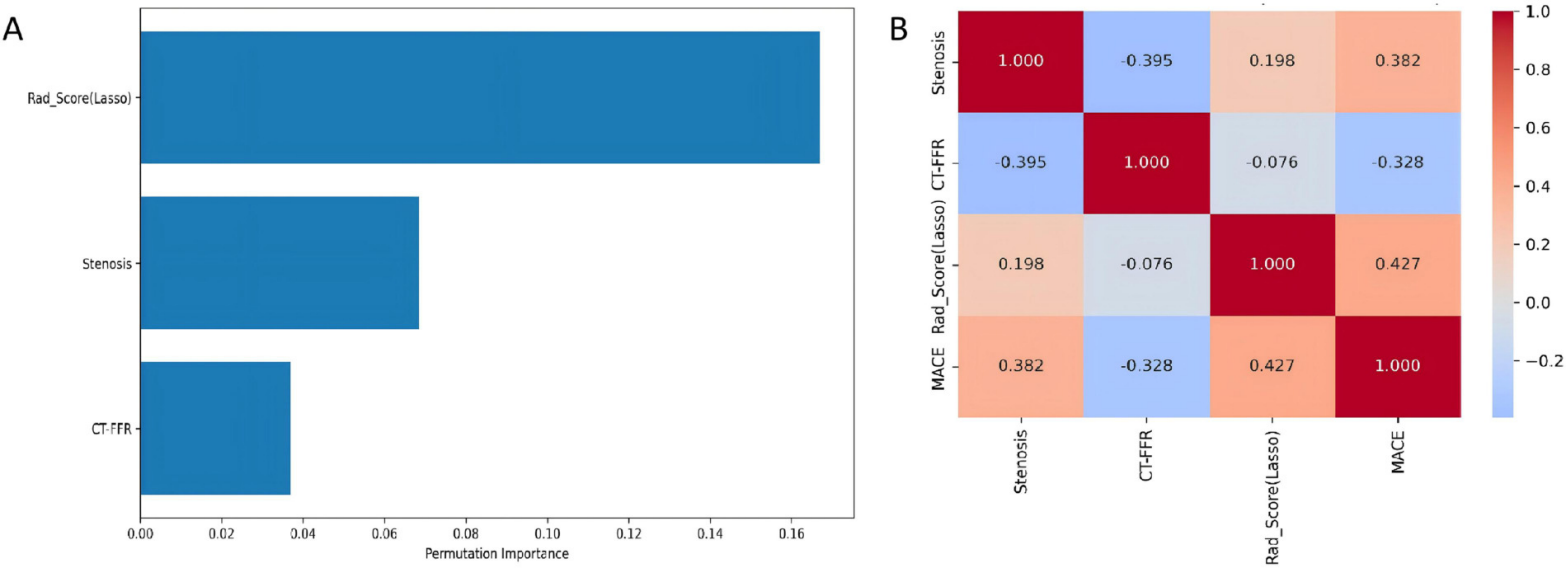
Feature importance analysis and correlation matrix for the optimal MACE prediction model. **(A)** Feature Importance for GP_Combined; **(B)** Feature Correlation Matrix. MACE, major adverse cardiovascular events; CT-FFR, CT-derived fractional flow reserve.

To enhance the clinical interpretability of the predictive models, the best-performing Combined Model 2 was further analyzed using permutation importance to assess feature contributions. Features were ranked in descending order based on their impact on model performance, with Rad-score emerging as the most influential predictor of MACE (Figure 5A). A Spearman rank correlation matrix was computed to assess monotonic associations among continuous predictors and binary MACE outcome. As shown in Figure 5B, Rad-score exhibited the strongest positive correlation with MACE occurrence (r = 0.427, *p* < 0.001), followed by stenosis severity (r = 0.382, *p* < 0.001); CT-FFR showed a moderate negative correlation (r = −0.328, *p* < 0.001). All correlations remained statistically significant after Bonferroni correction for multiple testing (*p* < 0.001). These findings suggest that the Rad-score, derived from PCAT radiomics features, captures additional phenotypic information beyond conventional anatomical and hemodynamic parameters, and may reflect underlying biological processes such as perivascular inflammation or plaque instability. However, further histopathological and mechanistic studies are warranted to elucidate the exact biological correlates of the Rad-score.

## 4 Discussion

This study investigated the predictive value of culprit vessel PCAT radiomics features and CCTA-derived functional parameters for MACE in patients with CAS. The results identified gender, culprit vessel stenosis severity, and CT-FFR as independent predictors of MACE. Both the CCTA-derived model, which incorporated stenosis severity and CT-FFR, and the combined model, which additionally included the Rad-score, demonstrated predictive value for MACE. Notably, the inclusion of radiomics features significantly enhanced the model's ability to predict adverse outcomes in CAS patients.

Studies have demonstrated that when the stenosis severity of a single coronary artery ranges from 70% to 99% and the patient is in a hypermetabolic state, CFR exhibits a significant reduction (14). In contrast, when coronary stenosis is less than 70%, CFR decreases exponentially with the progression of stenosis (15). Previous research has established that a coronary FFR ≤ 0.80 holds substantial clinical significance in predicting MACE and guiding revascularization strategies (16–18). Moreover, integrating the criteria of stenosis ≥ 70% and CT-FFR ≤ 0.80 into the assessment framework can notably enhance the prognostic stratification capability for patients with CAD (19). However, these prior studies relied on threshold-based criteria to determine the presence of coronary stenosis and insufficient flow reserve, which may compromise data granularity and result in information loss. In this study, instead of performing group-based classification of coronary stenosis severity and CT-FFR, we conducted quantitative measurements and analyses of these two parameters. Using these CCTA-derived indices, we developed a clinical prediction model for MACE. Our findings revealed statistically significant differences in coronary stenosis severity and CT-FFR between the MACE-negative and MACE-positive groups (*p* < 0.05). Furthermore, the prediction model constructed with these two parameters demonstrated a certain capacity to predict MACE occurrence in CAS patients. These results further validate that, regardless of whether threshold values are met, the exacerbation of coronary stenosis and the decline in flow reserve both carry predictive value for MACE in CAS patients.

As an inflammatory marker of the coronary arteries, PCAT is closely associated with coronary inflammation and the occurrence of MACE (20). Previous studies by Yang et al. (21) and Nishihara et al. (22). have demonstrated that elevated PCAT attenuation in patients with CAD is significantly correlated with MACE incidence. However, this metric only reflects the average level of PCAT attenuation and fails to capture the latent information embedded in CCTA images. The advent of radiomics enables the extraction of subvisual features from imaging data and their transformation into quantitative parameters, thereby facilitating precise disease assessment (23). A single-center retrospective study conducted by Zhan et al. (24) compared a PCAT radiomics model, a PCAT attenuation index model, and a CCTA-derived model. The findings indicated that the CCTA-based PCAT radiomics model is an effective tool for predicting MACE in patients with angina pectoris, and its predictive performance significantly outperforms that of the PCAT attenuation index model. Another study similarly confirmed that PCAT radiomics can substantially improve the long-term prediction of MACE compared to clinical scores, conventional CCTA, and PCAT attenuation (25). This study selected seven radiomics features (including four first-order features and three texture features), and a Rad-score was ultimately derived as the integrated radiomics feature. This Rad-score was combined with the stenosis severity of the culprit vessel and CT-FFR values to construct a combined prediction model. Following the inclusion of the Rad-score variable, the combined model exhibited significant improvements in all performance metrics. Permutation importance analysis of the combined model revealed that the Rad-score exerted the greatest influence on the model's predictive outcomes, indicating that PCAT radiomics provides incremental value for predicting MACE in CAS patients. Additionally, a statistically significant difference in Rad-score was observed between the MACE-negative and MACE-positive groups (*p* < 0.05), suggesting a potential association between the PCAT radiomics phenotype and the stability of coronary atherosclerotic plaques. Notably, the weak correlation between the Rad-score and CCTA-derived parameters in this study suggests that the Rad-score may reflect a microscopic dimension of plaque biological behavior that is independent of macroscopic morphology. Furthermore, given the relatively small sample size of this study, machine learning classifiers suitable for small-sample scenarios were employed. The core advantages of SVM lie in their structural risk minimization principle and maximum-margin classification mechanism, which enable them to maintain favorable generalization performance even with limited sample sizes (26). GPR is inherently suited for small-sample learning, as its model complexity is regulated by covariance functions, rendering it less prone to overfitting (27). The application of small-sample machine learning classifiers enhances the credibility of the study and validates the feasibility of PCAT radiomics in assisting MACE prediction.

Our study has several limitations. First, the measurement of FFR in this study was not validated against the invasive FFR gold standard. However, prior research has established the high accuracy of CT-FFR (28). Second, this study only explored the predictive value of stenosis severity and CT-FFR for MACE in patients with CAS, without incorporating plaque characteristics or compositional analysis to assess their impact on MACE occurrence. Third, this study was a single-center retrospective investigation. Future prospective studies incorporating samples from multiple centers are warranted to further validate the predictive utility of the model. Finally, the definition of MACE in this study included readmission for unstable angina with objective evidence of ischemia, which is broader than traditional definitions that often require revascularization. While this approach captures a wider spectrum of clinically relevant events, it may introduce heterogeneity. We did not perform a sensitivity analysis excluding patients with unstable angina alone; therefore, the impact of this broader definition on model performance remains uncertain. Future studies should consider such sensitivity analyses to confirm the robustness of our findings.

In summary, the combined model integrating PCAT radiomics features with clinical and imaging parameters offers incremental predictive value for MACE in patients with CAS. These findings provide valuable imaging-based evidence to support clinical prognosis assessment and inform therapeutic decision-making.

## Data Availability

The original contributions presented in the study are included in the article/supplementary material, further inquiries can be directed to the corresponding author.
