# Supplementary material for: Pericoronary adipose tissue radiomics enhances prediction of major adverse cardiovascular events beyond CCTA-derived functional parameters in coronary atherosclerosis
Source: Front Cardiovasc Med. 2026 May 12;13:1833189. doi: 10.3389/fcvm.2026.1833189 (PMC13201203; doi:10.3389/fcvm.2026.1833189)
Supplement: Supplementary file 1 [file Datasheet1.pdf]

Supplementary TABLE 1 Data preprocessing parameters, model hyperparameter settings, and custom algorithms used in model construction.

Category	Parameter/ Function Name	Value or Implementation Logic
Data Reading and Feature Selection	Clinical Model Feature Set	Stenosis, CT-FFR
	Combined Model Feature Set	Stenosis, CT-FFR, Rad_Score(Lasso)
	Outcome Variable	MACE
Model Hyperparameter Grid	SVM Parameter Search Space	C: [0.1, 1, 10, 100]<br>gamma: [0.001, 0.01, 0.1, 1]<br>kernel: ['rbf']
	GPR Parameter Search Space	kernel: [1.0 × RBF(0.5), 1.0 × RBF(1.0), 1.0 × RBF(2.0)]
Cross-Validation Settings	Data Splitting Strategy	StratifiedKFold(n_splits=5, shuffle=True, random_state=42)
Custom Evaluation Functions	calculate_metrics	Computes sensitivity, specificity, accuracy, precision, F1 score, AUC, and Brier score
	calculate_auc_ci	Calculates standard error of AUC using DeLong's method, constructs 95% confidence interval based on normal distribution: $CI = AUC \pm z_{0.975} \times SE_{AUC}$
	delong_test	Computes Z-statistic based on the covariance matrix of structural components: $Z = \frac{AUC_1 - AUC_2}{\sqrt{Var(AUC_1 - AUC_2)}}$
Decision Curve Analysis (DCA)	plot_decision_curve	Net benefit calculation formula:<br>$NB = \frac{TP}{N} - \frac{FP}{N} \times \frac{p_t}{1-p_t}$
	Reference Strategies	Treat All: $NB = \text{Prevalence} - (1-\text{Prevalence}) \times \frac{p_t}{1-p_t}$<br>Treat None: $NB = 0$
Model Interpretability Analysis	Permutation Importance	permutation_importance(..., scoring='roc_auc', n_repeats=10)
	Calibration Curve	calibration_curve(..., n_bins=10, strategy='quantile')

HDL, high-density lipoprotein; CT-FFR, CT-derived fractional flow reserve; SVM, support vector machine; MACE, major adverse cardiovascular events; GPR, Gaussian process regression; AUC, the area under the receiver operating characteristic curve;
